# Treatment Adherence and Secondary Prevention of Ischemic Stroke Among Discharged Patients Using Mobile Phone- and WeChat-Based Improvement Services: Cohort Study

**DOI:** 10.2196/16496

**Published:** 2020-04-15

**Authors:** Yuanjin Zhang, Dongsheng Fan, Hong Ji, Shudong Qiao, Xia Li

**Affiliations:** 1 Neurology Department Peking University Third Hospital Beijing China; 2 Information Management and Big Data Center Peking University Third Hospital Beijing China; 3 Neurology Department Peking University Shougang Hospital Beijing China

**Keywords:** stroke, secondary prevention, WeChat, self-monitoring

## Abstract

**Background:**

Real-world studies have indicated that adherence is important for guaranteeing medication effectiveness. Few studies have tested the feasibility and efficacy of WeChat-based improvement services, via mobile phone, in secondary prevention-specific follow-up among discharged stroke patients.

**Objective:**

We evaluated a quadruple-domain, WeChat-based service for ischemic stroke secondary prevention designed to improve treatment adherence of discharged patients. This service focuses on sending reminders for drug use, blood pressure recording, and glucose recording; it also records medication use. We compared the endpoint event rate between WeChat self-monitoring and traditional monitoring.

**Methods:**

A cohort study was used to determine the feasibility of a physician-assisted, WeChat-based improvement service and follow-up self-monitoring platform for the secondary prevention of ischemic stroke. The platform was developed by the Peking University Third Hospital based on the information-motivation-behavioral skills model. The overall adherence rate was calculated as the proportion of medication doses verified via uploading. The ischemic endpoint event rate and medication noncompliance rate were compared between traditional prevention monitoring and WeChat self-monitoring. Factors influencing adherence were summarized.

**Results:**

The 1-year follow-up event rate of the WeChat self-monitoring group was 11.9% (12/101), which was less than that of the traditional group (21/157, 13.4%). Compared with the traditional group, the risk ratio of the WeChat group was 0.983 (95% CI 0.895-1.080); this difference was not noted to be significant. The 1-year medication noncompliance ratio tended to be lower in the WeChat monitoring group (3/101, 3.0%) than in the traditional group (11/157, 7.0%; χ^2^=1.9, *df*=1, *P*=.16). Of the platform registry participants, 89.7% (210/234: 167 hospital-based and 43 community-based participants) adhered to inputting information into WeChat for 8-96 weeks. The average adherence time was 16.54 (SD 0.80, range 2-24) months. The average decrease in adherence was 4 participants (1.1%) per month. Being a member of a community-based population was an influencing factor for good adherence at the 2-year follow-up (OR 2.373, 95% CI 1.019-5.527, *P*=.045), whereas transient ischemic attack was an influencing factor for poor adherence at the 2-year follow-up (OR 0.122, 95% CI 0.016-0.940, *P*=.04).

**Conclusions:**

Use of WeChat self-monitoring showed a trend of increasing medication compliance and decreasing ischemic endpoint event rate compared with traditional monitoring. However, there were ceiling effects in the outcomes, and a relatively small sample size was used. Male participants displayed better adherence to WeChat self-monitoring. The community-based population displayed good adherence when using WeChat self-monitoring.

**Trial Registration:**

ClinicalTrials.gov NCT02618265; https://clinicaltrials.gov/ct2/show/NCT02618265

## Introduction

Stroke is one of the most common causes of death and long-term disability worldwide and in China [1-3]. Recurrent ischemic stroke accounts for nearly 17.7% of strokes in China according to the China National Stroke Registry [4]. Secondary prevention of ischemic stroke, including medical therapy and healthy lifestyle control, has been demonstrated from quantitative development and qualitative change for effectively preventing recurrent stroke or ischemic events. The combination of antiplatelet agents, statins, and antihypertensive agents is an effective therapeutic strategy and is recommended by various guidelines [5-8]. Good compliance of continuous monitoring to ensure that blood pressure, blood glucose, and lipids—particularly low-density lipoprotein cholesterol—reach therapeutic standards is essential for effective medication treatment to obtain prevention benefits. However, a real-world study has indicated that adherence is important for guaranteeing medication effectiveness [9]. Several studies have demonstrated that nearly 30% of patients failed to follow doctors’ instructions continuously [10,11]. Because of distinctions in the concept of health, incomplete health education, and unbalanced distribution of medical service resources, secondary prevention is not necessarily conducted by each institution continuously and may even be terminated by the patient. Thus, self-management directly supervised by hospital researchers after discharge is a potentially feasible and effective method for ensuring medication compliance and follow-up of endpoint events. It is widely recognized that using mobile phones can improve outreach and interactions with populations in need for health promotion and disease prevention [12,13]. WeChat is a simple, widespread communication app. Moreover, WeChat is cost-effective, less resource intensive, and more accessible than other communication apps; thus, enhancing adherence of prevention strategies seems feasible. The authors reviewed emerging technologies and methods that show promise in supporting and tracking medication compliance, then brought these together into a WeChat-based, follow-up self-monitoring platform that provided improvement services and was specific to ischemic stroke secondary prevention. The platform is part of the Peking University Third Hospital’s (PUTH) Health Service Account—the official WeChat account of PUTH—Department of Neurology follow-up domain (ie, the name of the platform's gateway). Few studies have tested the feasibility of this system as a prevention-specific follow-up approach among discharged stroke patients. We hypothesized that WeChat-based adherence monitoring, endpoint submission, and stroke-specific self-management will improve medication compliance rates and decrease recurrent stroke events. The objectives of this study were to (1) quantify the degree to which discharged participants adhere to prescribed modules of uploading medication adherence information and outcomes, (2) compare the event rate between traditional and WeChat adherence monitoring, and (3) explain longitudinal adherence rates.

## Methods

### Participants

Participants were recruited at three offline sites. The PUTH and the Peking University Shougang Hospital (PUSH) enrolled 300 acute ischemic stroke patients. The inclusion criteria of the hospital-based population were as follows:

1. Older than 18 years of age and can read or write.
2. Diagnosis of cerebral infarction with evidence from computed tomography or magnetic resonance imaging without coma—score of 0-25 on the National Institutes of Health Stroke Scale—and anterior (ie, internal carotid, anterior cerebral artery, and middle cerebral artery) circulation ischemia.
3. Diagnosis of transient ischemic attack (TIA) (ie, anterior circulation).
4. Had large vessel atherosclerotic and small vessel disease subtypes according to the Trial of Org 10172 in Acute Stroke Treatment (TOAST) criteria.

The exclusion criteria of this population were as follows:

1. Dementia.
2. Evidence of hemorrhage on computed tomography or magnetic resonance imaging.
3. Hematological disorders.
4. Any clinically relevant arrhythmia on admission, including atrial fibrillation.
5. Any major concurrent illness, including severe cardiovascular disease, liver or renal failure, and malignancies.
6. Fever, hypoxia, alterations in consciousness, or any relevant hemodynamic compromise on admission.
7. Any sensitivity to aspirin or clopidogrel.
8. Any other doctor-defined criterion as not suitable for enrollment.

All patients who were admitted to the neurology ward received therapeutic treatments according to the American Heart Association and American Stroke Association 2013 guidelines for acute ischemic stroke administration and the 2014 guidelines for ischemic stroke secondary prevention. All participants who met the inclusion criteria gave informed consent before enrollment and received routine health education about secondary prevention while being discharged. The inclusion criteria for adherence verification for the community-based population at high risk of stroke in the Huayuanlu Community Healthcare Service Center were history of stroke or TIA with no restriction of duration; the exclusion criteria were the same as those used for the hospital-based population.

### WeChat-Based Improvement Services and Self-Monitoring Platform for Discharged Patients

We developed WeChat-based, medication-related modules regarding the need for compliance. This was in accordance with the clinician’s recommendations for the patient and based on the individual medications and needs of the patient; thus, the app could function as a *personal trainer*. These modules provide people with timely reminders, according to their medication input dosage and frequency information; this occurs after collecting the uploaded prescription for secondary stroke prevention, which can be reinput and changed by the participant in accordance with the clinician’s prescription modifications. The individual’s characteristics (eg, disease-related records and monitoring-parameter levels) were recorded daily, automatically and manually. After certifying identification and receiving permission, the participating user was able to access the main menu of the WeChat-based platform and insert her or his information. The platform services were free for participants.

The PUTH Health Service Account, Department of Neurology follow-up domain, based on WeChat, created four modules and four items on the interface of the WeChat patient terminal. The modules emphasized the self-administration of prevention- therapy adherence, as explained by the designers and researchers. The construction and content of the four modules are described as follows:

1. Drug-use reminders: these provided therapeutic drug types, drug doses, original instructions for administration, and real-time modifications. The service platform automatically sent reminder messages according to a pre-established, inputted time point; feedback messages of “have taken medicine,” “have not taken medicine,” or no feedback were recorded by the central server.
2. Blood glucose recording: the module provided a recording option for glucose level (mmol/L) as well as a testing time and time point recording option that the patients could select; time point options included early morning, before breakfast, after breakfast, before lunch, after lunch, before evening dinner, after evening dinner, and before sleep. The interface of this module also provided a link for viewing the history record and the most recent blood glucose record.
3. Blood pressure recording: systolic blood pressure, diastolic blood pressure, heart rate, and measurement time option could be recorded. The interface of this module also provided a link for viewing the history record and the most recent blood pressure record.
4. Medication administration recording: the medication type and name, the period in which it was taken, and the condition in which it was taken could be reviewed and modified in this module.

**Figure 1:**
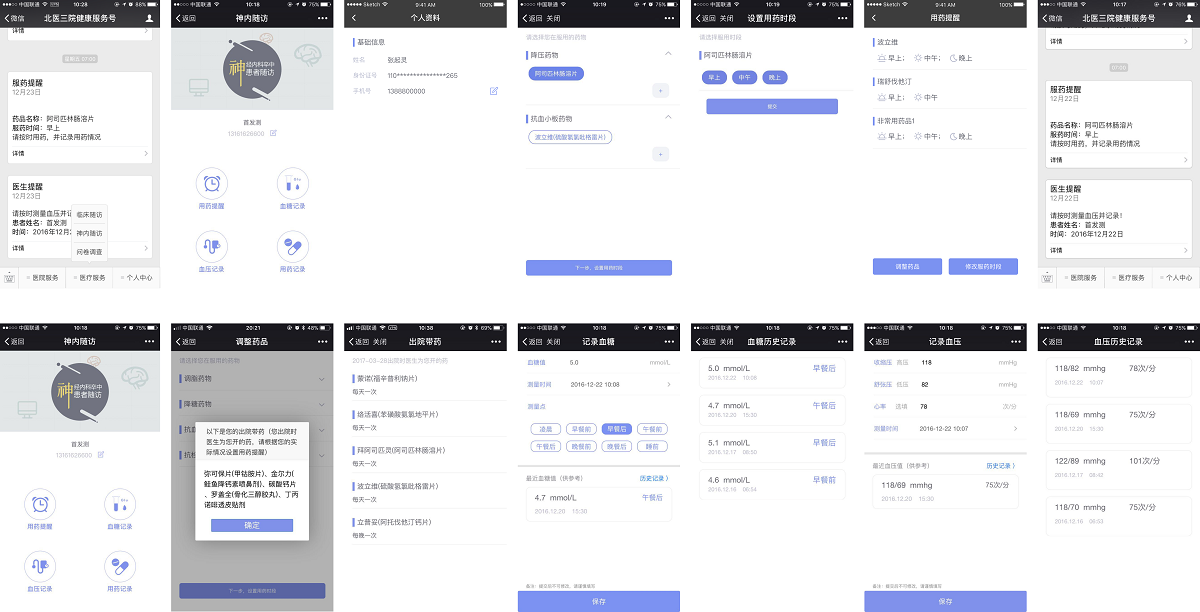
Screenshots of the WeChat-based self-monitoring program for prevention of secondary ischemic stroke, prevention-specific improvement services, and follow-up (Peking University Third Hospital, Health Service Account, Department of Neurology follow-up domain). The Web-based doctor terminal was included.

The four WeChat-based services included the following:

1. Operating guide: instruction video that included steps on how to set up the medication reminder, how to complete the medication record, and how to view the medication record, as well as textual descriptions.
2. Event reporting: provided a recent event option, including no special event to report, where situation options were categorized as all is normal and not going to hospital or normal clinician visit and no special event; disease attack, including cerebral infarction, intracranial hemorrhage, transient ischemic attack, and cardiovascular disease; and other situation, which the patient could enter via text.
3. Discharge medication recording: another special route for discharged patients to enter their medication dosages and frequencies.
4. Message consultation: provided a real-time communication window between the researchers and participants; the text characters were limited by the WeChat app to 20 words for each question (see Figure 1). The communication dialog box between the researchers and participants was monitored as a pilot test, since we received content irrelevant to clinical practice and medication.

**Figure 2:**
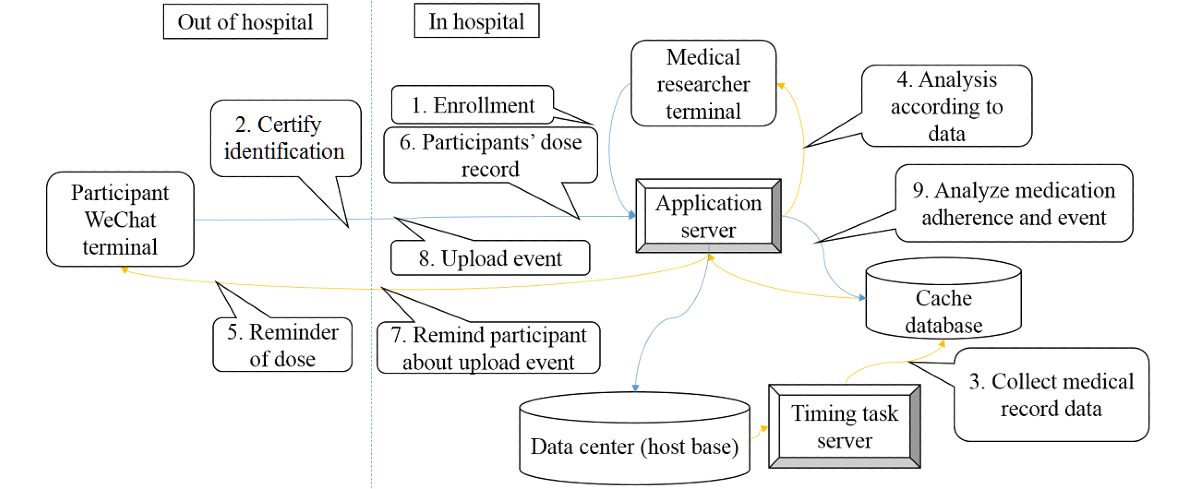
Information-interaction processing diagram of the WeChat platform.

**Figure 3:**
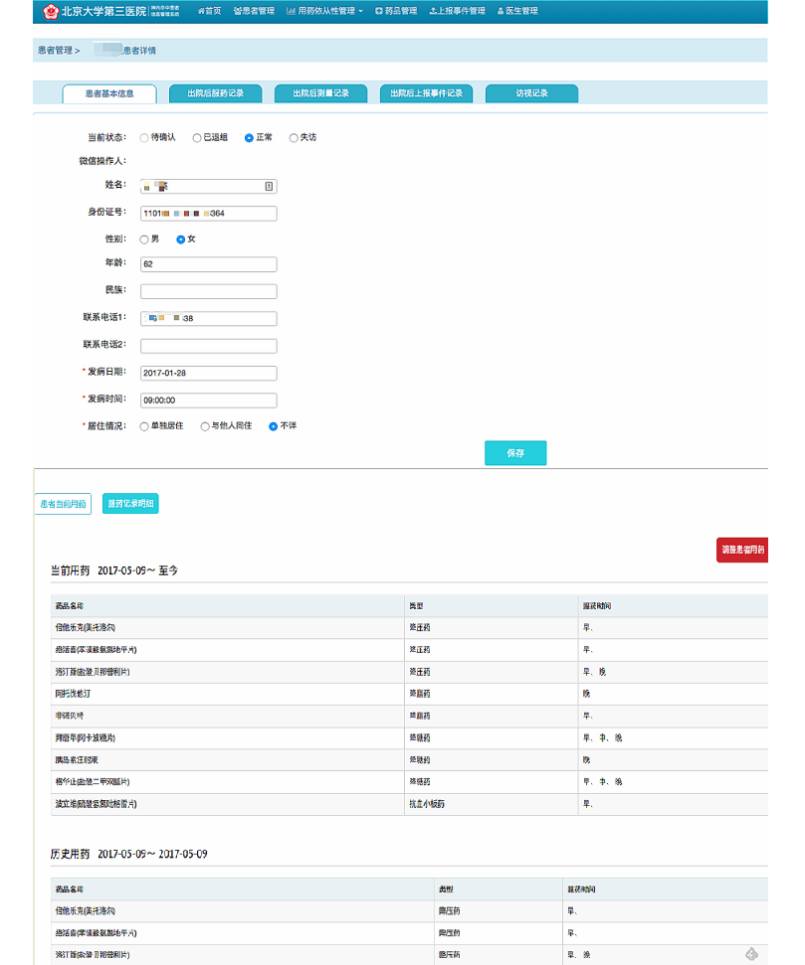
Synchronous web terminal of the WeChat information platform part 1.

**Figure 4:**
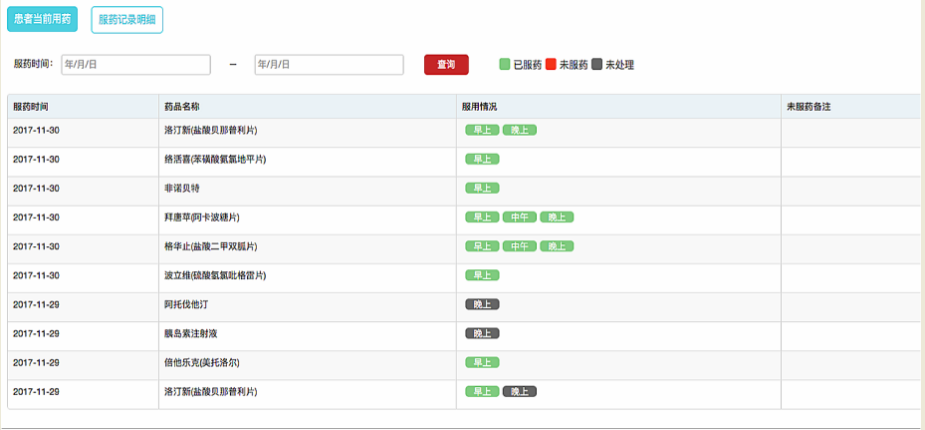
Synchronous web terminal of the WeChat information platform part 2.

In the doctor management website terminal (ie, the patient information management system from the Department of Neurology), every participant has a record identification number. The administrative medical record information was transferred to the website. Meanwhile, the website included the following four modules:

1. Patient information: the demographic and clinical information was synchronized with the hospital administration clinical records. The medication records, as well as the blood pressure and glucose measurements obtained via the WeChat terminal, could be viewed here.
2. Compliance indication: the number of 1-week and 2-week periods of noncompliance were highlighted, and the patients’ information could be obtained through this module.
3. Endpoint report information: patient-reported data in the WeChat terminal or manually inputted researcher follow-up data were shown, whereas only events defined by the researchers’ telephone interviews were analyzed.
4. Medication management: the medication compliance of every dose that ought to have been taken could be observed in this module. All the potential medications were listed for the patients to be able to see their options. Figures 2 to 4 show the theoretical framework of the platform.

### The Process for Implementing Monitoring

To ensure compliance of the demonstrated prevention elements, health education for secondary prevention began when the patients received antiplatelet agents, antihypertension agents, and statins in the stable disease period. Before discharge, on-site health education lectures and an introduction to the WeChat online interaction-monitoring platform were provided for each participant. The WeChat platform introduction, health education, and doctor recommendations were provided by telephone interview once every 3 months during the follow-up period if the participants had not received and adhered to the monitoring. The researchers logged in to the doctor terminal periodically to identify noncompliance; if noncompliance was identified, they contacted the participant to remind them to participate on the WeChat platform, routinely assessed follow-up endpoint event data, and investigated the reasons for nonadherence. The interactive communication via WeChat lasted 24 months and was facilitated and improved via modification from the academic members, based on participants’ feedback, until the final version was deemed sufficient. During this process, some irrelevant information was identified and feedback was provided regarding symptom descriptions. Additionally, during special political periods, services were restricted and were temporarily replaced by manual secondary prevention outpatient services.

### Research Design

**Figure 5:**
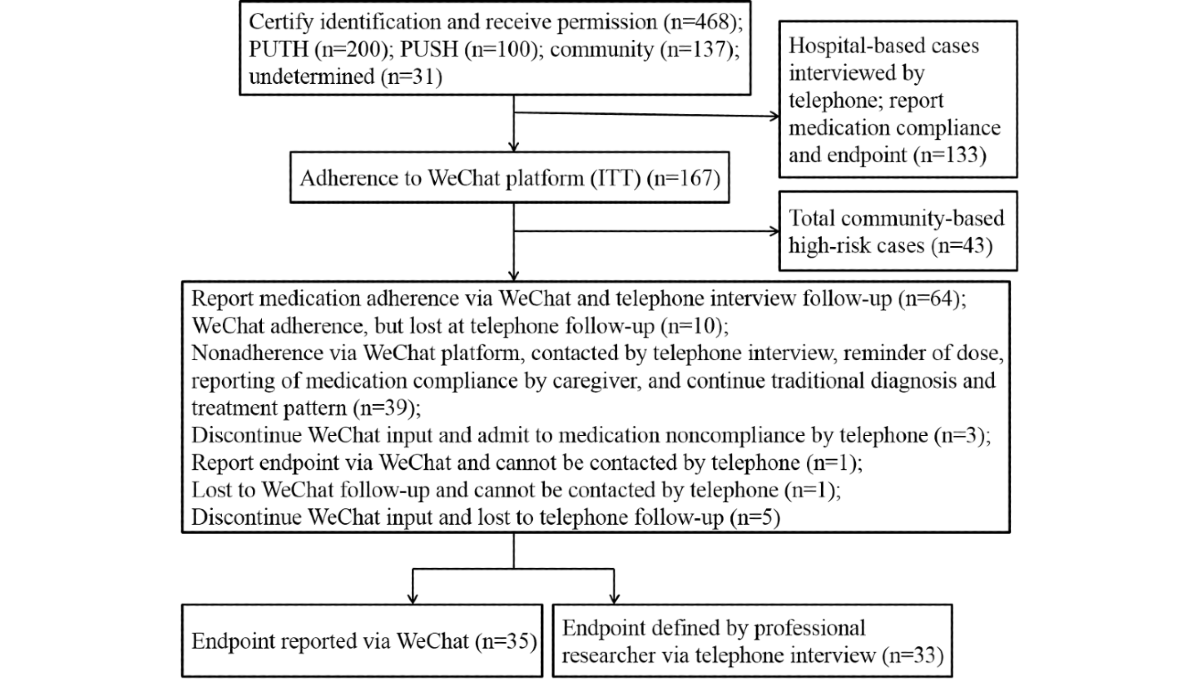
Flowchart of the study design. PUSH: Peking University Shougang Hospital; PUTH: Peking University Third Hospital; ITT: intention-to-treat.

This study was designed as a cohort study to examine the efficacy, feasibility, and acceptability of a WeChat-based service that was based on an information-motivation-behavioral skills model. The study protocol was approved by the Ethics Committee of the PUTH (2013-144) and PUSH (IRBK-2017-033-01), and participants gave written informed consent for participation. This study enrolled participants from September 2016 to September 2017. We compared the endpoint event rate between traditional prevention monitoring (ie, outpatient clinic visit, including health education and medication prescription) and WeChat monitoring (ie, medication adherence reminders, according to participants’ prescriptions, plus traditional monitoring). The flowchart of the study design is shown in Figure 5. A total of 2 years of follow-up data were collected to analyze WeChat platform adherence and related factors.

### Pilot Study Adherence Parameters and Outcomes

Compliance, or adherence, to a medication is defined as the extent to which participants continuously take all secondary preventive medications as prescribed at hospital discharge, except if their health care provider instructs them to stop taking a medication [14]. Compliance to treatment was evaluated by the number of days that the drug was taken. For participants whose medical records could be attached, we calculated the prescription dosage and cycle to determine the rate of compliance. For residents who were outside the hospital region after discharge, we investigated the adherence rate by telephone communication. Not adhering to the medication as instructed for 2 weeks was defined as noncompliance. Recurrent events included ischemic stroke, intracranial hemorrhage, transient ischemic attack demonstrated by imaging examination, and cardiovascular disease. The recurrent event outcomes were applied in the analysis. The loss to follow-up was recorded. WeChat endpoint events reported by the participants themselves were defined and classified through telephone interviews with the researchers and through medical records.

### Statistics

The data were analyzed using SPSS Statistics for Windows, version 22.0 (IBM Corp), and the figures were created using SPSS and Prism 5.0 (GraphPad). The feasibility analysis was conducted as intention-to-treat, based on the participants' recruitment. The risk ratio was calculated with SPSS and the recurrent event rate was compared between the WeChat self-monitoring group and the traditional group. The Kolmogorov-Smirnov test was used to determine the normality of the data. Normally distributed descriptive data were reported as mean and SD, and skewed distributions were reported as median with 25th and 75th percentiles (ie, quartiles). Demographic characteristics were compared using an independent *t* test for normally distributed continuous variables, based on the baseline data of the group with good adherence and the group with poor adherence. The nonparametric Mann-Whitney U test was used for continuous variables with skewed distributions, and the χ^2^ test was used for categorical variables. Trends and bias analysis was described using a Prism scatter graph and bar graphs. A logistic regression and a survival analysis were calculated using SPSS. A two-sided *P* value of .05 was considered statistically significant.

## Results

### Baseline Characteristics and Selection Bias Analysis

**Figure 6:**
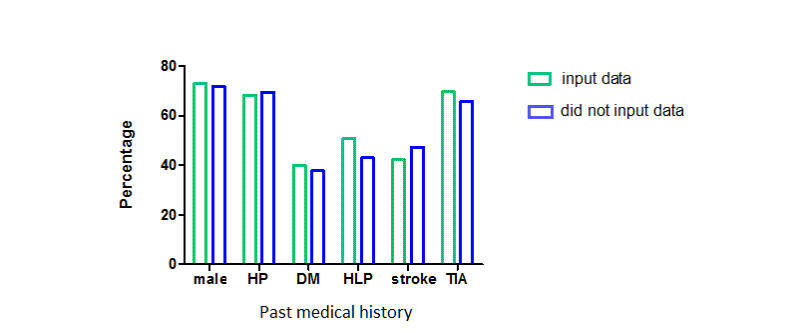
Selection bias analysis of the WeChat platform registry population. DM: diabetes mellitus; HLP: hyperlipidemia; HP: hypertension; TIA: transient ischemic attack.

A total of 468 participants (PUTH, 200/468, 42.7%; PUSH, 100/468, 21.4%; Huayuanlu Community Healthcare Service Center, 137/468, 29.3%; and undetermined, 31/468, 6.6%) were registered on the platform. Of those participants, 234 (50.0%) had used the platform at least once and had input data since enrollment. There were no statistically significant differences between the participants who did or did not input data (see Figure 6 and Table 1).

**Table 1:** Selection bias analysis of participants who did or did not input data into the WeChat platform registry.

Participant characteristic		Registry population		Statistic	*P* value		
		Input data	Did not input data				
**Demographic characteristic**							
	Age (years), mean (SD)	59.95 (14.42)	62.34 (11.67)	t_220_=-1.925		.06	
	Gender (male), n/N (%)	171/234 (73.1)	168/234 (71.8)	χ^2^_1_=0.1		.84	
**Medical history, n/N (%)**							
	Hypertension	160/234 (68.4)	163/234 (69.7)	χ^2^_1_=0.1		.84	
	Diabetes	84/210 (40.0)	85/225 (37.8)	χ^2^_1_=0.2		.69	
	Hyperlipidemia	107/210 (51.0)	97/225 (43.1)	χ^2^_1_=2.7		.10	
	Stroke history	97/229 (42.4)	110/232 (47.4)	χ^2^_2_=2.1		.35	
	Transient ischemic attack	147/211 (69.7)	148/225 (65.8)	χ^2^_1_=0.8		.41	

**Figure 7:**
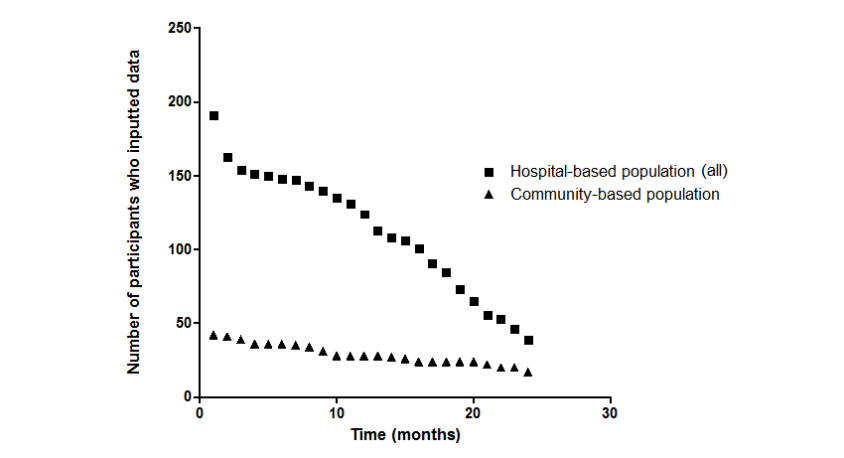
Participants' usage of the WeChat-based modules.

Within the research period, 167 hospital-based participants inputted data into WeChat after a 2-month learning and adaption period (see Figure 7). The *event report* domain was used by 13.9% (14/101) of participants until the 1-year follow-up, with endpoint data from the hospital-based population. The *drug use* module was used by 124 participants at 1 year, while 23 participants did not complete the telephone follow-up at 1 year. The *blood pressure* module was used by 90% (61/68) of participants with hypertension, and the application time (ie, the time that the participants actively used WeChat) was 14.18 (SD 9.04) months. The *glucose* module was used by 66% (44/67) of participants with diabetes, and the application time was 13.47 (SD 8.37) months.

Subjects were enrolled at the PUTH via verbal notification during discharge, followed by a text message, telephone introduction, and self-study of the platform instructions. Enrollment at the PUSH included bedside education and instruction within the administration period and self-study of the platform instructions. Enrollment of the community-based population included collective mission education, face-to-face instructions, and self-study of the platform instructions.

### Analysis of Medication Noncompliance: Follow-Up by Telephone and Reasons

The mean compliance time based on the inputted data, update of the platform vision based on participant feedback, and participant feedback regarding the WeChat platform’s drug use module showed that patients complied with antihypertension drugs, antiplatelet drugs, statins, and hypoglycemic drugs for an average of 15.52 (SD 7.24), 14.78 (SD 7.45), 15.42 (SD 7.11), and 19.05 (SD 6.72) months, respectively. At the 1-year telephone interview and medical record follow-up, 3.0% (3/101) of patients receiving WeChat monitoring and 7.0% (11/157) of patients receiving traditional monitoring admitted to self-determined medication noncompliance because they had no symptoms, did not understand the effectiveness of the medication, or did not realize the importance of medication compliance. The chi-square was 1.9 (*df*=1, *P*=.16). Overall, there was less nonadherence in the WeChat monitoring group than in the traditional monitoring group.

### Endpoint Recurrent Event Analysis

There were no statistically significant differences in age and gender between the WeChat self-monitoring group and the traditional monitoring group: WeChat group, 58.1 (SD 14.8) years of age versus traditional group, 60.1 (SD 15.4) years of age, t_28.5_=-0.999, *P*=.32; WeChat group, 22/101 (21.8%) males versus traditional group, 29/157 (18.5%) males, χ^2^_2_=0.4, *P*=.55.

According to the telephone interviews and endpoints identified by the researchers’ investigation of the medical records, the rate of loss to follow-up of the total hospital-based population was 14.0% (42/300). A total of 101 participants in the WeChat self-monitoring group and 157 participants in the traditional monitoring group could be contacted.

**Figure 8:**
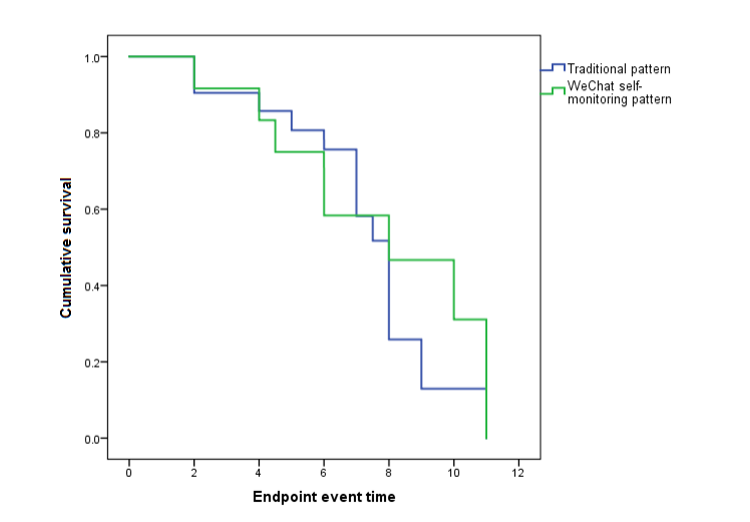
Survival functions: Kaplan-Meier analysis of the rate of endpoint events during the 1-year follow-up.

The 1-year follow-up event rate, as defined by the researcher, of the WeChat self-monitoring group was 11.9% (12/101), which was less than that of the traditional monitoring group (21/157, 13.4%). Compared with the traditional group, the risk ratio of the WeChat group was 0.983 (95% CI 0.895-1.080), which was not statistically significant. The mean endpoint event time of the WeChat self-monitoring group was 7.04 (SD 2.85) months, whereas that of the traditional group was 6.74 (SD 2.35) months. Figure 8 shows the survival curve of the event (log-rank test, *P*=.49).

There was no statistically significant difference in adherence time to the WeChat platform between the endpoint group (defined by WeChat self-report) (mean 17.8 [SD 7.5] months) and the nonendpoint group (mean 15.5 [SD 7.1] months) (*P*=.16), where endpoint was defined by the WeChat platform as reported by participants. Similarly, there was no statistically significant difference in adherence time between the endpoint group (mean 16.3 [SD 11.9] months) and the nonendpoint group (mean 16.3 [SD 6.7] months) (*P*=.99), where endpoint was assessed via telephone interview and medical record.

### Feasibility and Acceptability Parameters

**Figure 9:**
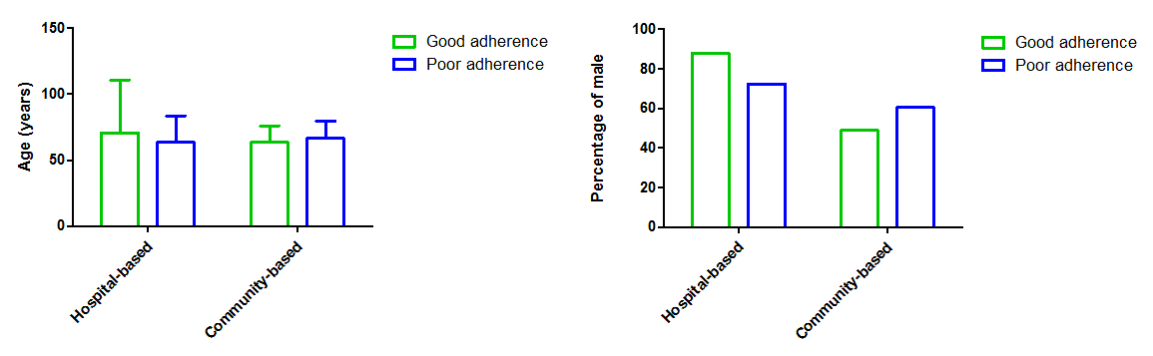
Demographics of the participants with good and poor adherence.

The differences between patients with good adherence and poor adherence were examined. Using each study site’s mean adherence time as the cutoff value (PUTH, 11.8 months; PUSH, 13.88 months; and community center, 16.63 months) with denoising, we summarized the valid data from 115 hospital-based and 43 community-based users with good adherence as well as 185 hospital-based and 94 community-based users with poor adherence. No significant difference in age was found between the patients with good adherence and those with poor adherence; however, more male participants adhered to inputting information into the WeChat platform (see Figure 9 and Table 2).

**Table 2:** Demographics of participants with good versus poor adherence in each population.

Demographic and population		Group with good adherence	Group with poor adherence	*P* value
**Gender (male), n/N (%)**				
	Hospital-based population	101/115 (87.8)	134/185 (72.4)	.002
	Community-based population	21/43 (49)	57/94 (61)	.17
**Age (years), mean (SD)**				
	Hospital-based population (good adherence n=115; poor adherence n=185)	70.8 (40.0)	63.6 (20.0)	.07
	Community-based population (good adherence n=43; poor adherence n=94)	63.5 (12.4)	66.5 (13.1)	.20

The number of participants who adhered decreased gradually each month (see Figure 7). The average adherence period was 16.5 (SD 0.8) months (range 2-24 months). The mean decrease in adherence was 4 participants (1.1%) per month. The slope for the decrease in adherence during the first year of follow-up (mean 4.0 [SD 0.2] cases/month) was significantly less than the slope during the second year of follow-up (mean 7.2 [SD 0.3] cases/month) (*P*<.001). The slope for the decrease in adherence of the hospital-based population (5.8 [SD 0.2] cases/month) was significantly greater than that for the community-based population (1.0 [SD 0.0] cases/month) (*P*<.001). A logistic regression showed that there were no risk factors for compliance during the first year (site: OR 1.529, 95% CI 0.906-2.579, *P*=.11; resident location: OR 2.014, 95% CI 0.648-6.262, *P*=.23; age: OR 0.979, 95% CI 0.948-1.011, *P*=.20; gender: OR 0.980, 95% CI 0.453-2.120, *P*=.96; stroke history: OR 0.979, 95% CI 0.948-1.011*, P*=.20; and TIA: OR 1.956, 95% CI 0.468-2.387, *P*=.90). Although community-based population status (OR 2.373, 95% CI 1.019-5.527, *P*=.045) and TIA (OR 0.122, 95% CI 0.016-0.940, *P*=.04) were factors that influenced 2-year compliance, other potential factors showed no statistical significance (resident location: OR 2.295, 95% CI 0.616-8.454, *P*=.22; age: OR 1.004, 95% CI 0.964-1.046, *P*=.85; gender: OR 1.529, 95% CI 0.567-4.121, *P*=.40; and stroke history: OR 1.277, 95% CI 0.575-2.840, *P*=.55).

### Reasons for WeChat-Based Nonadherence According to the Telephone Interview Follow-Up

The telephone interview had a loss to follow-up rate of 14.0% (42/300) among the hospital-based population, mostly because the telephone numbers left during the hospitalization period changed or belonged to people who were not the caregivers. The analysis of the reasons for WeChat nonadherence in the hospital-based population at the 1-year follow-up showed that 11.3% (34/300) of participants or caregivers had difficulty learning to use WeChat or did not use the app. Additionally, 1.7% (5/300) of the participants lacked an internet connection while traveling, and 31.7% (95/300) had a caregiver who provided reminders for medication, regularly sought medical services from a local hospital, and did not intend to participate in WeChat monitoring.

## Discussion

Previous studies have shown that an important explanation for medication noncompliance is lack of knowledge about reasons for adherence [15], dosages, and time of administration for the medication. In general, reasons for noncompliance reported by hospital-based neurological disease patients included modifications to the medication (41.7%) and forgetting to take the medication (33.2%) [16]. For patient-related factors, behavior approaches include either the use of aids, such as pill organizers, medication calendars, and blister packs, or active family involvement. We found an elevated level of medication compliance related to stroke secondary prevention among the WeChat monitoring group, which indicates the advantage of a WeChat reminder and confirms that mobile technology is potentially a promising means for improving health care.

The decreasing endpoint event rate reported in the WeChat monitoring group also indicated that recurrent events were delayed compared with traditional monitoring: the follow-up survival curve at 7 months after enrollment showed a better event rate among the WeChat group than among the traditional monitoring group. We attributed this to the accumulation effect of secondary prevention.

In this manuscript, we report that the innovative, stroke-specific, WeChat self-management program was well-received by participants at a local site as a useful supplement to medication adherence. The rate of decreasing adherence in inputting WeChat data was lower in the community-based population than in the hospital-based population; therefore, being a community-based participant was an influencing factor for good compliance in this study. This finding can be interpreted in that patient flow varies in different types of health institutions. Traditionally, discharged ischemic stroke patients undergo subsequent visits to the outpatient department of the hospital, community health care center, or local hospital of their choice near their residence, based on the available local medical resources. A proportion of participants with sudden-onset ischemic stroke would return to their local residential region after discharge. Moreover, participant-doctor relationships at community health service centers are often more interactive and friendly. The WeChat app has the advantages of being generalizable and able to reach more hospitals with the monitoring abilities and team needed for the high-risk stroke population. Promoting the use of local patient sites is an impactful and appropriate method to increase adherence.

We analyzed the factors that influenced self-monitoring adherence of the WeChat-based ischemic stroke secondary prevention, according to the slope parameters and the authors’ explanations. We found that WeChat self-management was better received by males, and we interpreted this finding to the stronger intrinsic motivation of male participants [17]. TIA is an influencing factor for poor adherence; except for selection bias, we think the lack of attention can be attributed to insufficient health education. The decrease in adherence rate was similar between the two hospital sites (PUTH slope: mean 3.0 [SD 0.2] cases/month; PUSH slope: mean 2.9 [SD 1.1] cases/month). The adherence rate was similar to other studies, in that it tended to be higher in the earlier months before dropping off over time [18]. The authors explained that there is a *learning effect*, in which participants would be encouraged to adhere only after having enough time to experience benefits from the WeChat-generated reminders and would then intend to benefit from adhering to the intervention method.

This study has several limitations. First, the lower WeChat adherence rate in our research was partially attributed to the fact that there was no statistically significant difference in the endpoint event rate between WeChat and traditional monitoring for secondary prevention, although the total recurrent ischemic stroke endpoint rate was less than that in previous reports. Second, the data from the blood glucose and blood pressure modules were not sufficiently transformed, since there was not enough input. The absence of sufficient risk-factor-control information, as well as relative communication via the WeChat terminal between doctor and participants, may have reduced the effectiveness of the secondary prevention. The research team members should provide ongoing support for the construction of the patient platform and doctor terminal; this would ensure delivery of the WeChat-based learning activities though WeChat videos and telephone calls during the implementation phase in order to avoid user fatigue over time. To our knowledge, the following are influencing factors of the feasibility of WeChat monitoring: performance expectancy (ie, improvements in ischemic stroke management and mind relaxation), effort expectancy (ie, ease of, or interest in, technical use), facilitating conditions (ie, availability of technical support and interactive doctor-participant communication), social influence (ie, support from caregivers), and habit (ie, the degree to which participating became a daily routine) [19]. Although we defined the endpoint of this study, we noticed that participants’ definitions of an event were not identical, so we struggled to upload an event as a potential influencing factor for adherence. Third, during the app progression and modification process, the module construction and improvements in function focused on ease of use, based on feedback from the participants. In future research, more effective types of therapy that could be conducted and popularized by the WeChat platform, or other attractive mobile health (mHealth) methods, should be introduced to increase the adherence rate.

Indeed, discharged patients are occasionally not compliant with their medication; however, we discovered that more discharged patients prefer face-to-face, traditional follow-up in clinical practice with a clinician. From the telephone follow-up, we found that daily, regular, medical practices of principal prevention strategies were well-adopted among our research population.

Since the diagnosis and treatment levels and medical resources are not identical across different hospitals and health service centers, the use of mHealth programs after discharge, such as WeChat platform monitoring, is convenient for patients and caregivers in both hospital-based and community-based populations. We should provide users with the best program, which is sustainable, dependable, and usable, in order to ensure that the self-monitoring service is well-received by the participants. The disadvantages of mHealth apps include incomplete network coverage and the participants’ or caregivers’ lack of internet technology skills regarding mobile phones and the WeChat terminal. Additionally, we expect that enabling innovative changes in secondary prevention practice to decrease the rate of recurrent stroke events will motivate learning. A two-arm, clustered, adaptability randomized controlled trial with lower loss to follow-up should be developed to demonstrate the effectiveness of mHealth programs.

## Appendix

Multimedia Appendix 1 CONSORT-eHEALTH checklist (V 1.6.1).

## Abbreviations

mHealth: mobile health
PUSH: Peking University Shougang Hospital
PUTH: Peking University Third Hospital
TIA: transient ischemic attack
TOAST: Trial of Org 10172 in Acute Stroke Treatment
