# Correction: Treatment Adherence and Secondary Prevention of Ischemic Stroke Among Discharged Patients Using Mobile Phone- and WeChat-Based Improvement Services: Cohort Study

**DOI:** 10.2196/19454

**Published:** 2020-04-29

**Authors:** Yuanjin Zhang, Dongsheng Fan, Hong Ji, Shudong Qiao, Xia Li

**Affiliations:** 1 Neurology Department Peking University Third Hospital Beijing China; 2 Information Management and Big Data Center Peking University Third Hospital Beijing China; 3 Neurology Department Peking University Shougang Hospital Beijing China

The authors of “Treatment Adherence and Secondary Prevention of Ischemic Stroke Among Discharged Patients Using Mobile Phone- and WeChat-Based Improvement Services: Cohort Study” (JMIR Mhealth Uhealth 2020;8(4):e16496) noticed several errors in their published article. The following corrections have been implemented:

In the Abstract, the sentence:

Of the platform registry participants, 88.9% (209/234: 167 hospital-based and 42 community-based participants) adhered to inputting information into WeChat for 8-96 weeks.

Has been corrected to:

Of the platform registry participants, 89.7% (210/234: 167 hospital-based and 43 community-based participants) adhered to inputting information into WeChat for 8-96 weeks.

This change does not impact the findings of the paper.

The following additional changes have also been made within the manuscript. The caption of Figure 3 has been corrected from “Untitled.” to:

Synchronous web terminal of the WeChat information platform part 1.

Figure 4 was formerly captioned:

Synchronous web terminal of the WeChat information platform.

This has been revised to:

Synchronous web terminal of the WeChat information platform part 2.

Figure 6 was formerly captioned:

Flowchart of the study design. PUSH: Peking University Shougang Hospital; PUTH: Peking University Third Hospital; ITT: intention-to-treat.

This has been revised to:

Selection bias analysis of the WeChat platform registry population. DM: diabetes mellitus; HLP: hyperlipidemia; HP: hypertension; TIA: transient ischemic attack.

Figure 7 was formerly captioned:

Selection bias analysis of the WeChat platform registry population. DM: diabetes mellitus; HLP: hyperlipidemia; HP: hypertension; TIA: transient ischemic attack.

This has been revised to:

Participants' usage of the WeChat-based modules.

Figure 8 was formerly captioned:

Participants' usage of the WeChat-based modules.

This has been revised to:

Survival functions: Kaplan-Meier analysis of the rate of endpoint events during the 1-year follow-up.

Finally, Figure 9 was formerly captioned:

Survival functions: Kaplan-Meier analysis of the rate of endpoint events during the 1-year follow-up.

This has been revised to:

Demographics of the participants with good and poor adherence.

The correction will appear in the online version of the paper on the JMIR website on April 29, together with the publication of this correction notice. Because this was made after submission to PubMed, PubMed Central, and other full-text repositories, the corrected article has also been resubmitted to those repositories.

